# Mitochondrial Adaptations Underlying Tetraploidization in Human Cancer, Fungal, and Yeast Models

**DOI:** 10.3390/biology15020181

**Published:** 2026-01-19

**Authors:** Mohamed Jemaà, Ameni Bedoui, Nihel Ammous, Ali Gargouri, Mohamed Guerfali

**Affiliations:** 1 Human Genetics Laboratory, Faculty of Medicine of Tunis, Tunis El Manar University, 5, Rue Hassouna Ben Ayed–1007 Bab Saadoun Tunis, Tunis 2092, Tunisia; 2 Neurophysiology, Cellular Physiopathology and Valorisation of Biomolecules Laboratory, Faculty of Sciences of Tunis, Tunis El Manar University, Tunis 2092, Tunisia; 3 Department of Biology, Faculty of Sciences of Tunis, Tunis El Manar University, Campus Universitaire El-Manar, 2092 El Manar, Tunis 2092, Tunisia; 4 Laboratory of Molecular Biotechnology of Eukaryotes, LMBE, Centre of Biotechnology of Sfax, P.O. Box 1177, Sfax 3038, Tunisia

**Keywords:** polyploidy, size, mitochondria, mtDNA, flow cytometry

## Abstract

Whole-genome duplication, or tetraploidization, occurs in cells, tissues, or entire organisms. In human cancers, tetraploidization promotes aneuploidy and genomic instability, accelerating tumor progression, metastasis, and drug resistance. These adaptations demand metabolic rewiring, including mitochondrial plasticity. Here, we investigate the relationship between mitochondrial quantity/activity, including the mitochondrial transmembrane potential, the intracellular calcium, and the oxidative stress in diploid versus tetraploid cancer cells (colon, sarcoma, liver) and fungal and yeast models (*C. albicans* diploid/tetraploid strains; *S. cerevisiae* haploid/diploid/tetraploid strains). We demonstrate that tetraploid cells, whether from human carcinomas or yeast, exhibit consistently enlarged cell size, elevated mitochondrial content, and heightened metabolic activity compared to diploids. Our findings underscore mitochondrial adaptation as a hallmark of tetraploidization, offering novel therapeutic targets for chromosomally unstable tumors.

## Simple Summary

In many types of cancer, cells double their entire set of chromosomes, a state known as tetraploidy. This change can accelerate the cancer’s growth, spread, and resistance to drugs. We wanted to understand how this chromosome doubling changes the cell’s mitochondria. We compared cells with a normal versus a doubled set of chromosomes from human cancers, yeast, and fungi. We found that in all cases, cells with doubled chromosomes were larger and contained more mitochondria, which were also more active. By identifying this shared trait, our research points to these adapted mitochondria as a potential new target for treating cancers with unstable chromosomes.

## 1. Introduction

Polyploidization plays a vital role in normal development, tissue homeostasis, and regeneration. It also drives evolutionary processes, such as adaptation and speciation [1]. Polyploidy naturally occurs at cellular or tissue levels. In humans, for instance, specialized polyploid cells facilitate tissue homeostasis and wound healing, as well as performing unique functions such as the production of platelets from polyploid megakaryocytes [2]. Essentially, polyploidy is accumulation of more than two sets of chromosomes, and it can result from cell fusion, endoreplication, or karyokinesis without cytokinesis [3]. However, polyploidization in humans can contribute to pathological conditions, particularly cancer [4], and is even correlated with aggressiveness [5] and metastasis [6].

The simplest form of polyploidy, tetraploidy (4n) is the condition of having twice as many chromosomes as their normal, diploid counterparts [7,8]. These cells can be found at a relatively high frequency in highly proliferating tissues like the placenta [9] or tumors, for instance [10].

The correlation between elevated proliferation and evolutionary processes, including migration and differentiation, is indicative of an enhanced metabolic rate for tetraploid cells. The hypothesis is that the cells that underwent endoreplication to become tetraploid will have doubled their molecular equipment, including their mitochondria. Evidence also suggests that cell size control is of significance. For example, hepatocytes have been demonstrated to remodel their metabolome and proteome as their size increases [11,12]. Experimentally, in their study, Michael C Lanz et al. showed that primary human fetal lung fibroblasts and hTERT-immortalized RPE-1 cells modulated their metabolism in relation to their size [13].

There is a redundant locution that has become a slogan for biology students to demonstrate the importance of mitochondria in cell physiology: *Mitochondria are the powerhouses of the cell*. Indeed, mitochondria are double-membraned, cytoplasmic organelles that contain their own genome, which encodes proteins that are critical for respiration. However, the majority of mitochondrial proteins are encoded by the nucleus [14]. Mitochondria are pivotal to cellular bioenergetics. These organelles are vital for the production of adenosine triphosphate (ATP) via oxidative phosphorylation, and they play a central role in the biosynthesis of macromolecules [15]. The mitochondrial architecture exhibits pronounced plasticity, with cell-type-specific structural and proteomic adaptations that support tissue-specific metabolic functions [16]. Mitochondria thus emerged as a key research focus across multiple disciplines due to their central role in diverse cellular processes. So, do tetraploid cells need more mitochondria to survive an enhanced metabolism and a different cellular function? [14,17].

Our work set out to analyze the quantity of mitochondria and their activity in diploid and tetraploid cells from three different cancer models: colon, sarcoma, and liver cancer. We also used yeast cells with haploid, diploid, and tetraploid status in addition to fungal diploid and tetraploid cells to address such a fundamental question. The main goal was straightforward: to confirm the correlation between cell ploidy and mitochondrial content and dynamics.

## 2. Materials and Methods

### 2.1. Cell Lines and Culture Conditions

Diploid and tetraploid clones derived from human colon carcinoma RKO cells [6] were maintained in McCoy’s 5A medium supplemented with 10% fetal calf serum (FCS), 10 mM HEPES buffer, and 1% penicillin/streptomycin. Diploid and tetraploid clones derived from malignant fibrous histiocytoma MFH152 (soft-tissue sarcoma subtype) and near-diploid and near-tetraploid clones derived from hepatocellular carcinoma HepG2 were grown in Dulbecco’s modified Eagle’s medium (DMEM) supplemented with 10% FCS and 1% penicillin/streptomycin [18,19] (all provided by Thermo Fisher Scientific-Gibco, St. Louis, MA, USA). Cells were routinely maintained at 37 °C under 5% CO_2_ and were seeded onto the appropriate supports 24 h before the beginning of experiments.

### 2.2. Yeast and Fungal Strains

Three *S. cerevisiae* strains differing by their ploidy were used in this study: the haploid strain W303-1B (α ade2, ura3, his3, trp1, leu2) [20]; the diploid WB111, constructed by crossing W303-1B by B111 (a his1 trp2, rho-) [21] and verified by growth on minimal media, growth on glycerol medium, and sporulation ability; and the tetraploid *S. cerevisiae* AOB strain, a commercial baker’s yeast provided by Bousalem Yeast Factories, Tunisia. In addition, two stable diploid and tetraploid *C. albicans* strains were also used.

### 2.3. Cytofluorometric Studies

Cytofluorometric acquisitions were performed by means of a FACSVerse cytofluorometer (BD Biosciences, San Jose, CA, USA). Data were statistically evaluated using the FSC Express 6 Flow software (De Novo Software, Pasadena, CA, USA). Only events characterized by normal forward scatter (FSC) and side scatter (SSC) parameters were gated for inclusion in the statistical analysis after exclusion of cell doublets.

*1- Quantification of Cell Size and Form*

Cells were collected and acquired using flow cytometry and the normal light scattering parameters of forward scatter (FSC) vs. side scatter (SSC). The dot plot was set to a linear scale.

*2- Cell Cycle Analysis*

For the assessment of cell cycle distribution, cells were collected, washed once with PBS, and then fixed by gentle vortexing in ice-cold 75% (*v*/*v*) ethanol for 30 s. After overnight incubation at −20 °C, samples were centrifuged, washed with PBS, and stained with 50 μg/mL PI in 0.1% (*w*/*v*) D-glucose in PBS supplemented with 1 μg/mL (*w*/*v*) RNase A (Sigma–Aldrich, St. Louis, MO, USA) for 30 min at 37 °C. Afterward, samples were incubated overnight at 4 °C before cytofluorometric analysis.

*3- Measurement of Mitochondria Accumulation*

For staining mitochondria, cells were harvested and collected with the culture medium and labeled for 45 min at 37 °C with 100 nM of MitoTracker Red MTR (ThermoFisher, Waltham, MA, USA) before FACS assessment.

*4- Measurement of Mitochondrial Transmembrane Potential* (Δψm)

To measure the mitochondrial transmembrane potential (ΔΨm), the cells, yeast, or fungi were collected in the culture medium and then labeled in a serum-free medium solution containing the ΔΨm-specific dye DiOC6(3) (40 nM) for 30 min at 37 °C prior to FACS analysis.

*5- Measurement of Intracellular Calcium Concentration*

To assess the intracellular calcium concentration, cells were collected, washed in PBS, and suspended in growth medium supplemented with 5 μM of the calcium tracker Fluo-3/AM (Biotium, Hayward, CA, USA). Cells were incubated at 37 °C for 30 min before calcium-dependent fluorescence intensity measurement.

*6- Measurement of Oxidative Stress Accumulation*

To quantify oxidative stress, cells were collected, washed, and suspended in PBS solution with 10 μM 2′,7′-dichlorodihydrofluorescein diacetate (DCFDA) (Sigma, Schnelldorf, Germany) to measure the reactive oxygen species (ROS). Cells were incubated at 37 °C for 30 min before cytofluorometric analysis.

### 2.4. Total Yeast DNA Extraction

The strains were cultured on YPG medium (1% yeast extract, 1% Bacto-Peptone and 2% glucose). Their total DNA was extracted with minor modifications [22]: 1 mL of cultured cells was transferred in an Eppendorf tube and centrifuged, and the pellet was suspended in Solution A containing 1 mL SCK (1 M sorbitol, 50 mM citric acid, 150 mM K_2_HPO_4_), 10 mM EDTA pH 8, 0.1% mercaptoethanol, and 10 units of zymolyase (Zymo Research, Irvine, CA, USA). The cell wall digestion by zymolyase was carried out at 37 °C for one hour. The spheroplasts are pelleted by centrifugation for 5 min at 5000 rpm and directly suspended in 400 μL of solution B (150 mM NaCl, 10 mM Tris pH 7, 5 mM EDTA pH 8, and 1% Sarkosyl). The viscous lysates were then twice extracted with an equal volume of Tris-saturated Phenol (Sigma–Aldrich). After centrifugation at 12,000 rpm for 5 min, the upper phase was then twice extracted with an equal volume of chloroform. The final upper aqueous phase was mixed with 1 mL ethanol and left at −20 °C overnight. The nucleic acids were then pelleted by centrifugation at 12,000 rpm for 10 min, dried, and suspended in 50 μL of bi-distilled water. The quantity of DNA was determined spectrophotometrically at 260 nm and their quality assessed through electrophoresis in agarose gel using TAE buffer.

### 2.5. DNA Digestion and Densitometry Analysis

10 μL of DNA were treated with the restriction enzyme HhaI in the recommended buffer O (Fermentas, Burlington, ON, Canada) for 3 h at 37 °C. Thereafter, the digested DNA was migrated in a 1% agarose gel under TAE buffer. After staining with ethidium bromide, the gel was photographed under UV light. The intensity of the bands was analyzed using Image J Version 1.51K software.

### 2.6. qPCR Assays and Determination of Mitochondrial DNA Copy Number

For each strain (tetraploid, diploid, and haploid), the qPCR reaction mixture contained 1 µL of total DNA as template, 2 pmol of each primer pair, and 5 µL of 2× TB Green Premix Ex Taq (Takara, Kusatsu, Japan) for a final reaction volume of 10 µL. Real-time amplification was performed on a Bio-Rad CFX96 system under the following cycling conditions: initial denaturation at 95 °C for 1 min, followed by 40 cycles of 94 °C for 10 s and 57 °C for 30 s. The specificity of the PCR products was verified by melting curve analysis from 65 °C to 95 °C, with a heating rate of 0.5 °C/s. All samples were run in triplicate (20 ng DNA/replicate), including nuclease-free water as a no-template control (NTC). Once normalized, the mitochondrial gene target Ct value was analyzed using the 2-Delta-Delta method to convert the Ct value to a linear form for analysis of gene expression and calculate the relative change in mitochondrial copy number. We followed the method of Maggo et al. [23] but we substituted the human nuclear (BECN1 and NEB) and mitochondrial genes (ND1 and ND6) gene with the following yeast ones: the nuclear genes were, respectively, TDH (triose-phosphate dehydrogenase) and ACT (actin), while the mitochondrial genes were SMITO (15S rRNA) and COB (cytochrome b). The nucleotide sequences of each pair of primers are indicated in Table 1. Briefly, Cts from the SMITO gene were subtracted from those of the TDH gene to obtain ΔCt1 (ΔCt1 = CtNuc1 − CtMito1), while COB Ct was subtracted from ACT Ct to obtain ΔCt2 (ΔCt2 = CtNuc2 − CtMito2); then, copy numbers were calculated based on the ΔCt of the matched mitochondrial and nuclear DNA Cts (N = 2ΔCt). The average of the different copy number results provides the final relative mtDNA copy number per sample.

### 2.7. Statistical Analyses

All experiments were performed and independently repeated at least three times. Results from diploid and tetraploid cells were randomly paired to compute differences and compared statistically using a two-tailed Wilcoxon–Mann–Whitney test with GraphPad Prism 10.6.1. Data are expressed as arithmetic means ± SEM.

## 3. Results

### 3.1. Tetraploid Cells Are Larger Then Diploid Cells and Contain More Mitochondria

**Figure 1:**
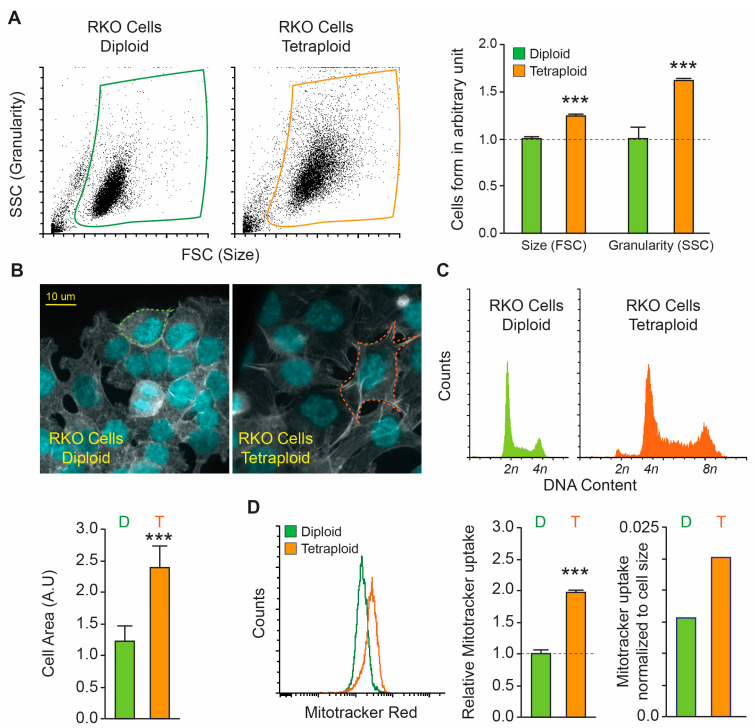
**Characterization of the size and form of diploid and tetraploid RKO cells.** (**A**) Flow cytometry dot plots showing the forward scatter (size) versus side scatter (granularity) parameters of diploid and tetraploid RKO cells, along with associated quantification data. (**B**) Cell area analysis. Representative micrographs of diploid and tetraploid RKO clones labeled with phalloidin (actin) and DAPI (DNA) are shown, with an interrupted line surrounding the cells for each condition. Scale bar = 10 μm. Quantitative data on cell area are displayed. (**C**) Flow cytometry histograms showing the diploid and tetraploid cell cycle. (**D**) Diploid and tetraploid cells were stained with MitoTracker Red (MTR) to quantify mitochondrial matrix. The histogram shows overlap between the diploid and tetraploid conditions, while the quantification reveals mitochondrial MTR uptake of the dye in both conditions, in addition to normalization to cell size in each condition. Diploid cells are labeled in green and tetraploid cells in orange. Data are reported as means ± SEM (*n* ≥ 3). *** *p* < 0.001 (Mann–Whitney test) compared with the diploid cells.

In order to perform our study, we used the human colon carcinoma RKO diploid and tetraploid clones that we previously generated [6,24]. We started by evaluating the clones’ size using flow cytometry and light scattering parameters forward scatter (FSC) vs. side scatter (SSC), and we found that tetraploid clones were significantly larger than diploid clones. The tetraploid cells were 1.16 times larger and 1.59 times more granular than the diploid cells (Figure 1A). In addition, when we observed the cells under a microscope, photographed them, and calculated their area using Image J, we found that the tetraploid cells were 1.95 times larger than the diploid cells (2387 ± 234 vs. 1223 ± 236 in arbitrary units) (Figure 1B). To corroborate the ploidy status of the clones, we performed a cell cycle analysis and confirmed the diploid vs. tetraploid karyotype (Figure 1C). These data were further confirmed using other cellular origins, namely diploid and tetraploid clones derived from malignant fibrous histiocytoma MFH152 (soft-tissue sarcoma subtype) [18] (Figure S1A,B) and near-diploid and near-tetraploid clones derived from hepatocellular carcinoma HepG2 [19] (Figure S2A).

We decided then to quantify the mitochondrial content of the cells and we performed a flow cytometry assay using MitoTracker Red, a mitochondrial potential-independent dye able to stain live mitochondria [25]. We found that tetraploid RKO clones contain 1.97 times more mitochondria than diploid ones, with a tracker uptake of 0.013 times cell size in diploids, compared to 0.021 times in tetraploids (Figure 1D).

### 3.2. Mitochondria Are More Functional in Tetraploid Cells

**Figure 2:**
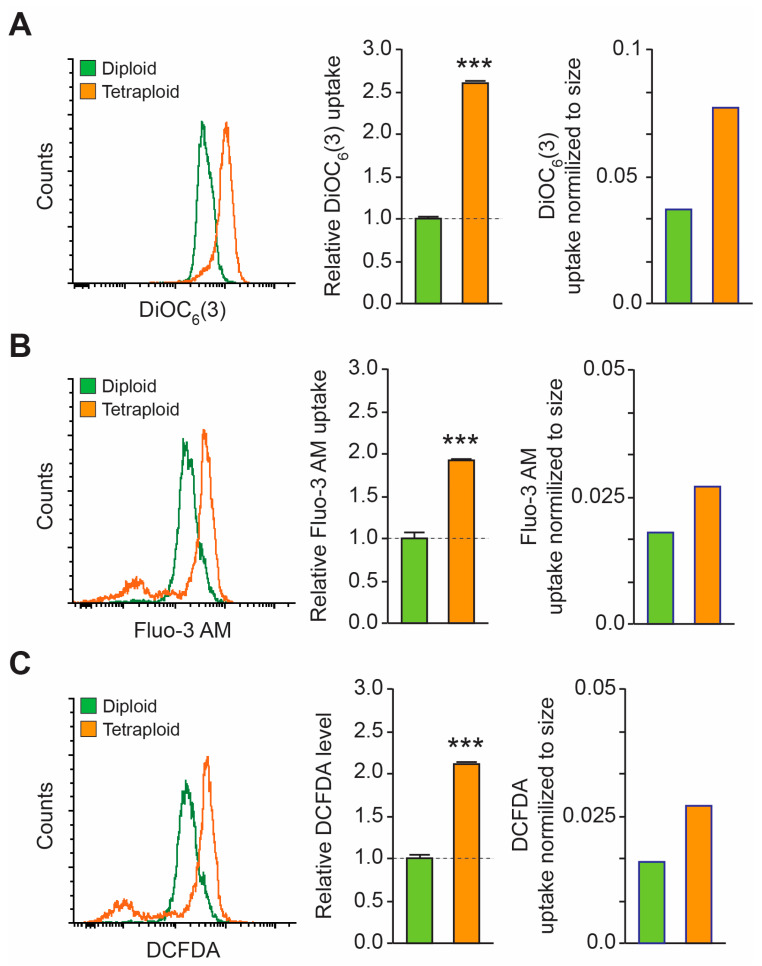
**Mitochondrial activity in diploid and tetraploid RKO cells.** (**A**) Diploid and tetraploid RKO cells were stained with the DiOC6(3) dye to quantify the mitochondrial transmembrane potential (Δψ_m) by flow cytometry. The histograms show overlap between the two cell types, while the quantification reveals mitochondrial uptake of the dyes in both conditions, in addition to normalization to cell size in each condition. (**B**) Diploid and tetraploid RKO cells were stained with the calcium dye Fluo-3 to quantify the cytosolic Ca^2+^ concentration by flow cytometry. The histogram shows overlap between the two cell types, while the quantification displays the calcium concentration in both conditions, in addition to normalization to cell size in each condition. (**C**) Diploid and tetraploid RKO cells were stained with the ROS sensor DCFDA to measure reactive oxygen species (ROS) and quantify oxidative stress by flow cytometry. DCFDA is non-fluorescent, but in the presence of ROS, it is oxidized and becomes green fluorescent. The histogram shows overlap between the diploid and tetraploid cells, while the quantification displays the presence of reactive oxygen species (ROS) in both conditions, in addition to normalization to cell size in each condition. Diploid cells are labeled in green and tetraploid cells in orange. Data are reported as means ± SEM (*n* ≥ 3). *** *p* < 0.001 (Mann–Whitney test) compared with the diploid cells.

In order to access mitochondrial activity in diploid vs. tetraploid clones, we first evaluated the mitochondrial inner transmembrane potential (Δψm) using the specific dye DiOC6(3) [26]. Indeed, the Δψm is a key indicator of the energetic state of the mitochondria, and thus its functions, especially the mitochondrial proton pumps, electrogenic transport systems, and the activation of the mitochondrial permeability transition [27]. We found that RKO tetraploid clones had more active mitochondria than diploid clones, with 2.6 times more dye uptake and a cell size normalization factor of 0.037 in diploid cells compared to 0.077 in tetraploid cells (Figure 2A). Similar data were also found in MFH152 (Figure S1C) and HepG2 clones (Figure S2B).

Cell signal transduction by calcium and reactive oxygen species (ROS) are also important markers of mitochondrial activity [28]. We assessed the intracellular calcium accumulation ([Ca^2+^]i) using the Fluo3 fluorescence Fluo-3 AM dye [29]. Tetraploid cells had 1.9 times more intracellular calcium than diploid cells, and a ([Ca^2+^]i)-to-cell-size normalization factor of 0.018 in diploid cells compared to 0.027 in tetraploid cells (Figure 2B). Similarly, utilizing the DCFDA sensor, we quantified the reactive oxygen species (ROS) in the cells. DCFDA is non-fluorescent, but in the presence of ROS, it is oxidized and becomes green fluorescent and measurable [30]. We found that there was 2.1 times more ROS activation in tetraploid cells than in diploid cells, and a ROS-to-cell-size normalization factor of 0.016 in diploid cells compared to 0.027 in tetraploid cells (Figure 2C).

### 3.3. Mitochondria Level Is Correlated with Ploidy in Yeast and Fungal Strains

**Figure 3:**
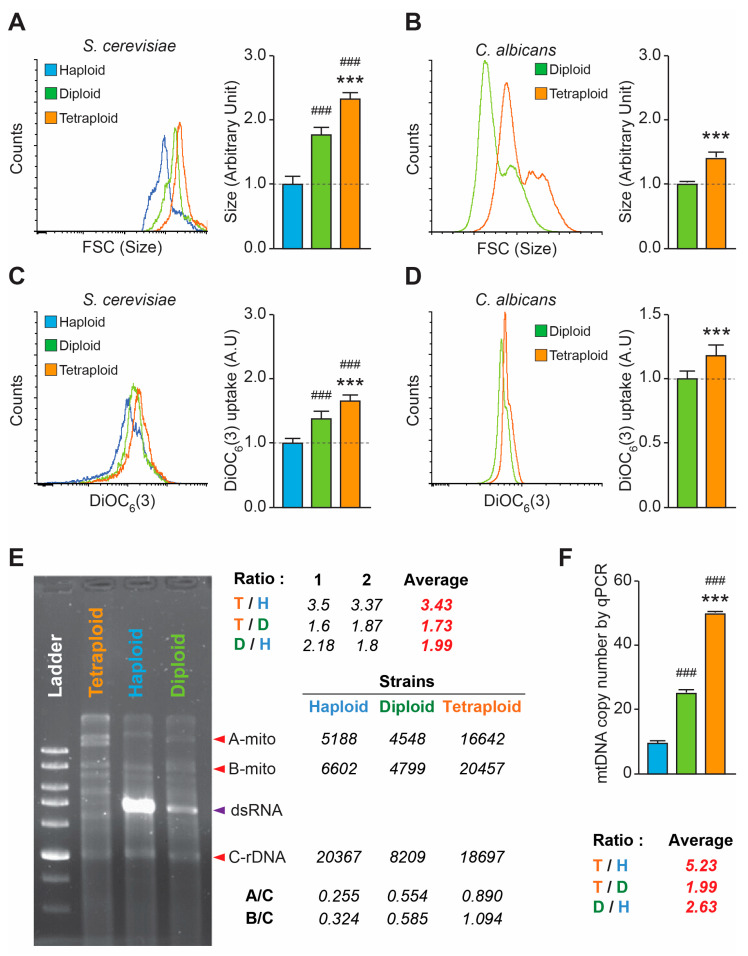
**Characterization of yeast and fungal strains by size and mitochondrial activity.** (**A**) Flow cytometry histograms showing the overlap of the forward scatter (size) parameters of haploid (labeled blue), diploid (labeled green), and tetraploid (labeled orange) *S. cerevisiae* yeast strains, along with the related quantification data normalized to the haploid strain. (**B**) Flow cytometry histograms showing the forward scatter (size) parameter overlaps of diploid (labeled green) and tetraploid (labeled orange) *C. albicans* strains, along with the related quantification data normalized to the diploid strain. (**C**) Haploid, diploid, and tetraploid *S. cerevisiae* yeast strains were stained with the dye DiOC6(3) to quantify the mitochondrial transmembrane potential (Δψ_m) by flow cytometry. The histograms show overlap between the haploid, diploid, and tetraploid strains, while the quantification reveals mitochondrial uptake of the dye in all conditions, normalized against haploid strain uptake. (**D**) Diploid and tetraploid *C. albicans* strains were stained with the dye DiOC6(3) to quantify the mitochondrial transmembrane potential (ΔΨ_m) by flow cytometry. The histograms show overlap between the diploid and tetraploid strains, and the quantification reveals mitochondrial uptake of the dye in all conditions, normalized against the uptake of the diploid strain. (**E**) The total DNA of haploid, diploid, and tetraploid *S. cerevisiae* yeast strains was extracted and treated with the HhaI restriction enzyme (which cuts at GCGC sites). The electrophoresis gel shows the intensities of several DNA bands, which were analyzed using Image J software. The mtDNA (poor in CG content) produces large fragments that migrate to the upper part of the gel (up to the dsRNA), while the nucDNA (rich in CG content) is digested into small fragments that migrate under the dsRNA. The ratio of two chosen mitochondrial bands, A and B, to the nuclear rDNA (C), gives an indication of the relative amount of mitochondrial DNA in the cell. Subsequently, the ratios 1 and 2 of several combinations (e.g., for T/H, these ratios are 1 = (A/C of T)/(A/C of H) and 2 = (B/C of T)/(B/C of H)) provide a more accurate comparison of the mitochondrial DNA content in different ploidy states. Thus, the average mtDNA ratios for the combinations tetraploid/diploid, tetraploid/haploid, and diploid/haploid are 1.99, 1.73 and 3.43, respectively. (**F**) Relative quantification of mtDNA copy number in haploid, diploid, and tetraploid *S. cerevisiae* yeast strains was performed using real-time PCR analysis and two mitochondrial genes (SMITO (15S rRNA) and COB (cytochrome b)), normalized to actin and triose-phosphate dehydrogenase, in addition to the ratio of the combinations tetraploid/diploid, tetraploid/haploid, and diploid/haploid (average mtDNA copy number of 2.63 in diploid compared to haploid, 1.99 in tetraploid compared to diploid, and 5.23 in tetraploid compared to haploid). Data are reported as means ± SEM (*n* ≥ 3). *** *p* < 0.001 (Mann–Whitney test) compared with diploid, and ^###^ *p* < 0.001 (Mann–Whitney test) compared with haploid.

In order to ask the fundamental question regarding the ploidy and the mitochondrial quantity and functionality, we decided to use additional models: fungal and yeast. Indeed, we took advantage of two *C. albicans* strains and three *S. cerevisiae* strains differing by their ploidy. The ploidy was verified by growing on minimal media, growing on glycerol medium, and sporulation ability. We first assessed the size of three *S. cerevisiae* strains (haploid, diploid, and tetraploid) and two *C. albicans* strains (diploid and tetraploid) using flow cytometry and light scattering parameters (forward scatter, FSC). We found that the diploid *S. cerevisiae* strain was larger than the haploid strain, but smaller than the tetraploid strain. Indeed, the FSC parameter was 1.38 times larger in the diploid strain than in the haploid strain, and 2.33 times larger in the tetraploid strain than in the haploid strain (Figure 3A). The tetraploid *C. albicans* strain was 1.4 times larger than the diploid strain (Figure 3B). Then, we assessed the mitochondrial inner transmembrane potential (Δψm) using the specific dye DiOC6(3), and found the same correlation regarding mitochondrial activity. In *S. cerevisiae*, haploid yeast had the weakest signal, followed by diploid yeast, with tetraploid yeast having the strongest signal (1.66 times higher DiOC6(3) uptake in tetraploid yeast strains compared to haploid strains, and 1.38 times higher in diploid strains compared to haploid strains) (Figure 3C). In the *C. albicans* strain, we found that the DiOC6(3) uptake in the tetraploid strain was 1.18 times higher than in the diploid strain (Figure 3D).

We then wanted to quantify the mitochondria in each *S. cerevisiae* strain to confirm our findings with human cells. To do so, we used mitochondrial DNA (mtDNA). The rationale behind this methodology is based on the fact that GCGC sites are very rare in yeast mtDNA, at around 17%, as opposed to yeast nuclear DNA (nucDNA), which is almost 38% GC-rich [31]. We extracted the entire DNA and digested it using the HhaI restriction enzyme, which cuts at GCGC sites. The result was that the digested mtDNA produced large fragments that migrated in an electrophoresis gel, while the nucDNA was digested into small fragments that smeared. It is also important to note the presence of killer dsRNA, which is a large double-stranded RNA that resists digestion. However, it is not present in all strains, only in haploid and diploid strains. When we ran an electrophoresis gel, the upper part consisted of mtDNA up to the killer dsRNA, while the nucDNA smear migrated below the killer dsRNA (Figure 3E). The electrophoretic profile enabled us to determine the ratio of the intensity of the mtDNA band, which migrates in the upper part of the gel (above the dsRNA), to that of the nucDNA band, which migrates in the lower part of the gel. We chose two mtDNA bands and one nucDNA band. Thus, the ratio of the two chosen mitochondrial bands (1 and 2) to the nuclear band gave us an idea of the relative amount of mitochondrial DNA in the yeasts. We found that there was 3.43 times more mtDNA in tetraploid yeast than in haploid yeast, 1.73 times more in tetraploid yeast than in diploid yeast, and 1.99 times more in diploid yeast than in haploid yeast (Figure 3E).

Following the same result, quantitative PCR analysis of the three strains revealed a clear proportional relationship between ploidy level and mitochondrial DNA (mtDNA) copy number. The tetraploid strain had the highest mtDNA copy number (mtDNAcn) with 49.7 copies, followed by the diploid strain with 25.03 copies and the haploid strain with 9.5 copies. We calculated the ploidy-related ratio and found that there was 5.23 times more mtDNA in tetraploid yeast than in haploid yeast, 1.99 times more in tetraploid yeast than in diploid yeast, and 2.63 times more in diploid yeast than in haploid yeast (Figure 3F).

## 4. Discussion

**Figure 4:**
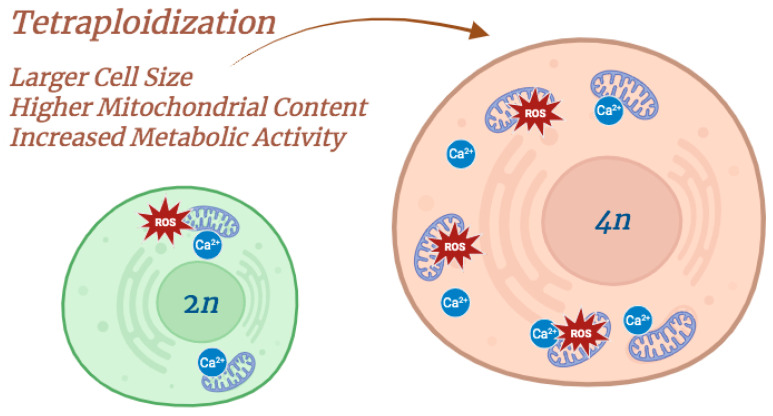
Graphical synthesis of high mitochondrial activity coupled with increased size in tetraploid cells. Created with BioRender.com/2025.

The present study aimed to provide further evidence in support of the hypothesis that polyploidization is correlated with mitochondrial activity. To this end, we employed a variety of cellular models, including human cancer cells, as well as fungal and yeast cells. We assessed the mitochondrial inner transmembrane potential (Δψ_m). Additionally, oxidative stress and the concentration of intracellular calcium were measured, and mitochondrial activity was quantified in correlation with cell size and ploidy (Figure 4).

Mitochondria have gained significant research interest across various disciplines, given their participation in numerous cellular processes. This study is a continuation of a project aiming to understand how changes in chromosome number and cell size can make cancer cells more aggressive. This includes how cancer cells can resist chemotherapy and radiotherapy and how they can spread [4,5,6,8,18,32,33,34,35,36].

Our cells that underwent whole-genome duplication and tetraploidy showed an increase in mitochondrial function that could potentially be exploited as a vulnerability. This aligns with conserved cellular responses to ploidy increase, where tetraploidization triggers proteome remodeling via the downregulation of mTORC1/S6K signaling, leading to sub-linear scaling of protein synthesis and ribosomal biogenesis. Consequently, the mitochondrial activity we observed may represent a critical compensatory or dysregulated component within this scaled anabolic program [37].

As our study found, tetraploid and, more generally, chromosomally unstable CIN cancer cells are specifically dependent on mitochondrial metabolism and should be hypersensitive to inhibitors and inducers of oxidative stress [38] and dysregulated mitophagy leading to accumulation of dysfunctional mitochondria [39].

The canonical view, established in models ranging from HeLa cells to budding yeast, holds that mitochondrial content scales proportionally with cell size to maintain a constant organelle-to-cytoplasm volume ratio [40,41]. However, the correlation of mitochondrial activity with size and ploidy remains a matter of debate. A recent study explored how metabolic rate relates to mitochondrial power and host cell size by assessing metabolic activity in approximately 170 unicellular eukaryotes. The findings revealed no definitive link between mitochondrial power and cell size, and the expected linear scaling of mitochondrial activity across cell sizes was not supported, *de facto* depending on cell type [42].

When focusing on human cells, another notable report demonstrated, using single-cell analysis, that mitochondrial function (but not content) peaks at intermediate cell sizes, creating a fitness optimum. Size control is orchestrated by growth factors and maintained via mevalonate-dependent mitochondrial dynamics, linking metabolic efficiency to proliferative capacity [12]. However, it was highlighted that the mitochondrial membrane potential changes in accordance with cell size, rather than cell cycle. By “cell cycle”, it should be noted that the focus is on the G2/M phase during mitosis, excluding tetraploidy resulting in endoreplication, for example. This may indicate a gap in the study’s scope [12]. This is further confirmed when the manipulation of hepatocyte size revealed a nonlinear relationship with mitochondrial respiration, where oxidative phosphorylation peaked at intermediate cell sizes [11]. This phenomenon may be indicative of an adaptive mechanism designed to minimize ROS accumulation within the liver’s toxic microenvironment [43]. Such adaptive downregulation of mitochondrial gene expression and function is a cell-autonomous response to large cell size, serving to uncouple growth from proliferation [11]. In the context of pathological tetraploidization in cancer, this homeostatic mechanism may be subverted or insufficient, leading to the metabolic imbalances we observe.

Another study demonstrated that polyploid prostate and mammary epithelial cells exhibit increased mitochondrial content and consequently higher ROS levels compared to diploid cells. Furthermore, when polyploidization was induced using Cytochalasin D (which blocks cytokinesis), researchers observed similarly elevated ROS levels and mitochondrial mass [44]. Also, hypoxia triggers polyploidization in cardiomyocytes, resulting in multi-nucleation and cellular hypertrophy. However, these enlarged cells display diminished ROS generation and apoptosis, mediated by upregulation of mitophagy, a key process in mitochondrial homeostasis [45].

In another study, murine decidual cells, abnormal uterine decidual tissue formations in non-pregnant mice, were found to comprise polyploid (both multi-nucleated or mono-nucleated) and non-polyploid populations. Polyploid cells displayed elevated mitochondrial mass and ATP production. Transcriptomic profiling (Affymetrix) and functional enrichment analysis implicated polyploidy-associated genes in metabolic and mitochondrial pathways [46].

Gregory J Thomson et al. showed in their paper that their tetraploid *C. albicans* cells displayed a 2.2-fold volume increase over diploids, concomitant with elevated MitoTracker Green fluorescence (reflecting higher mitochondrial mass) and ROS production suggesting that tetraploid metabolic activity is augmented by their larger size and expanded mitochondrial capacity [47]. It should be noted here that we did not observe the same phenomenon within our fungal cells when normalized to size.

*A contrario*, a recent and elegant study investigated the whole tetraploidization of *Caenorhabditis elegans* and its effect on the worm physiology and sensitivity to chemotherapeutics, including DNA-damaging drugs like cisplatin and doxorubicin. The study revealed that despite the presence of acquired resistance to DNA damage and apoptosis, no mitochondrial or metabolic differences were found between diploid and tetraploid *C. elegans* [48].

While mitochondrial parameters initially correlate with ploidy (tetraploidy), extreme enlargement activates mitophagy to limit ROS-induced damage. In cancer, this adaptation may drive tumorigenesis and malignant progression.

## 5. Conclusions

Our study establishes a clear relationship between cellular ploidy and mitochondrial activity across multiple model systems. We demonstrate that tetraploid cells, whether derived from human carcinomas (RKO, MFH152, HepG2), fungal (*C. albicans*), or yeast strains (*S. cerevisiae*), consistently exhibit increased cell size, mitochondrial content, and metabolic activity compared to their diploid counterparts. These findings imply that tetraploidization acts as a metabolic amplifier, potentially conferring survival advantages in stress conditions, including chemotherapy and radiotherapy, or acquiring extra capacity like enhanced metastasis. This may involve a disruption of the careful coordination between nuclear and mitochondrial DNA copy number, which is normally linked to cell volume via limiting nuclear-encoded factors [49]. Complementary studies are planned to confirm whether targeting ploidy-associated metabolic pathways could offer therapeutic opportunities in polyploidy-driven diseases.

## Figures and Tables

**Table 1:** Primers utilized, with two nuclear genes: TDH (triose-phosphate dehydrogenase) and ACT (actin) and two mitochondrial genes: SMITO (15S rRNA) and COB (cytochrome b); F: forward, R: reverse.

Gene	Primer Name	Sequence
TDH	TDH-F	TTGTTGACTTGACTGTCAAG
	TDH-R	AAGCCTTGGCAACATATTCG
ACT	ACT-F	ACACACGTGTTCCCATCGGT
	ACT-R	AAGAACTGGGTGCTCTTCTG
SMITO	SMITO-F	ACTAATATTTGTGCCAGCAG
	SMITO-R	AATCCGTTTCGCTACTCTAG
COB	COB-F	GATTCACCACAACCATCATC
	COB-R	CTTGGTGATCTATATGAACC

## Data Availability

If not available in Supplementary Figures, data available on request from the authors.

## Supplementary Materials

The following supporting information can be downloaded at: https://www.mdpi.com/article/10.3390/biology15020181/s1, Figure S1: Characterization of sarcoma diploid and tetraploid cell size, form and mitochondrial potential; Figure S2: Characterization of the size, form and mitochondrial potential of diploid and tetraploid liver cancer cells.
