# Supplementary material for: Mitochondrial Adaptations Underlying Tetraploidization in Human Cancer, Fungal, and Yeast Models
Source: Biology (Basel). 2026 Jan 19;15(2):181. doi: 10.3390/biology15020181 (PMC12837461; doi:10.3390/biology15020181)
Supplement: Supplementary file 1 [file biology-15-00181-s001.zip › biology-3818701-supplementary.pdf]

Supplementary Materials:

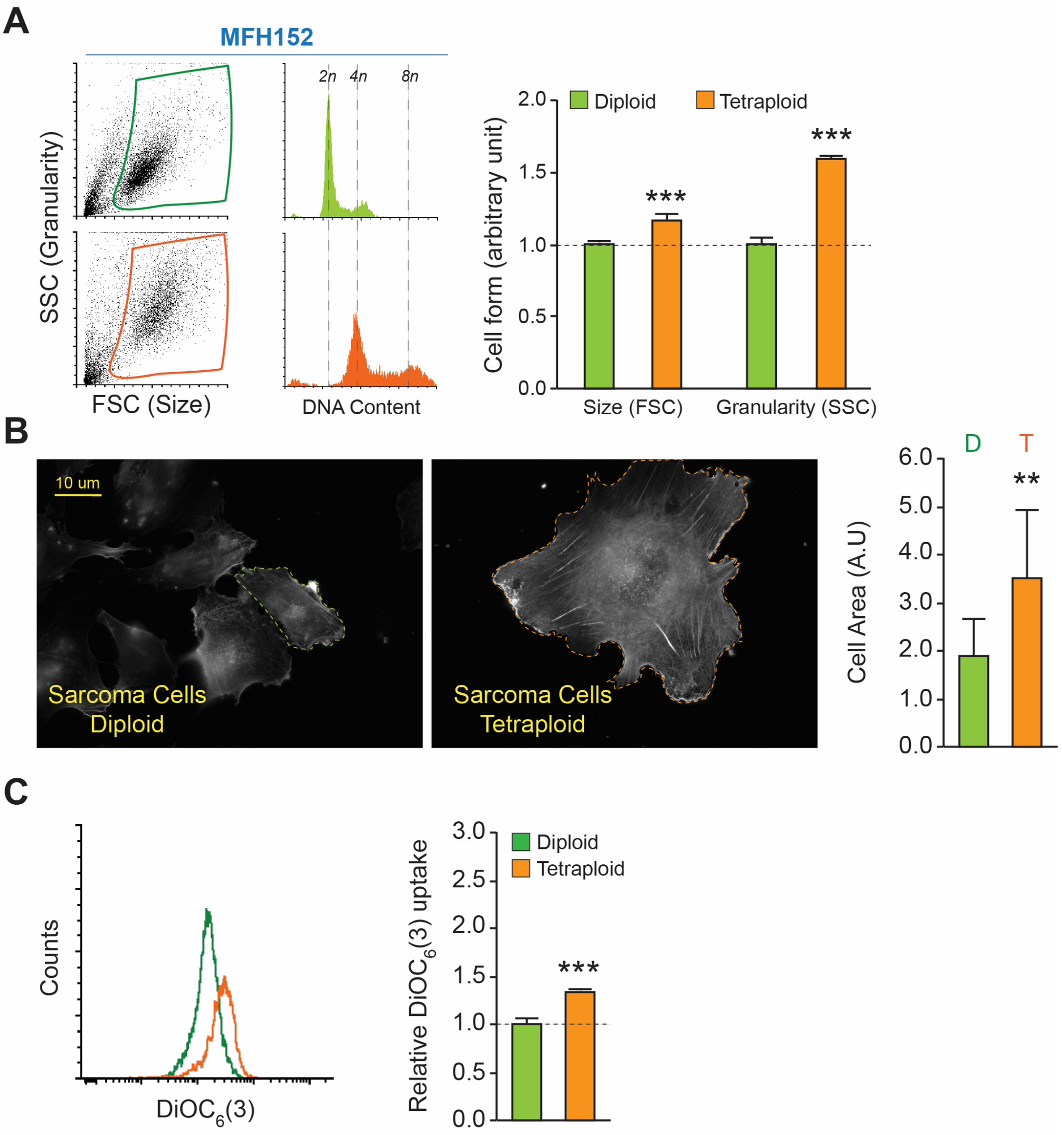

Figure S1: Characterization of sarcoma diploid and tetraploid cell size, form and mitochondrial potential. A. Flow cytometry dot plots showing forward scatter (size) versus side scatter (granularity) parameters and an associated histogram showing the cell cycle of diploid and tetraploid MFH152 cells. Related quantification data is also shown. **B.** Cell area analysis. Representative micrographs of diploid and tetraploid RKO clones labeled with phalloidin (actin) and DAPI (DNA) are shown, with an interrupted line surrounding each condition. Scale bar = 10 μm. Quantitative data on cell area are displayed. **C.** Diploid and tetraploid MFH152 cells were stained with the dye DiOC6(3) to quantify the mitochondrial transmembrane potential (Δψ_m) by flow cytometry. The histograms show overlap between the diploid and tetraploid conditions, while quantification reveals mitochondrial uptake of the dyes in both conditions. Diploid cells are labeled in green and tetraploid cells in orange. Data are reported as means ± SEM ($n \geq 3$). *** $p < 0.001$, ** $p < 0.01$ (Mann–Whitney test) compared with diploid cells.

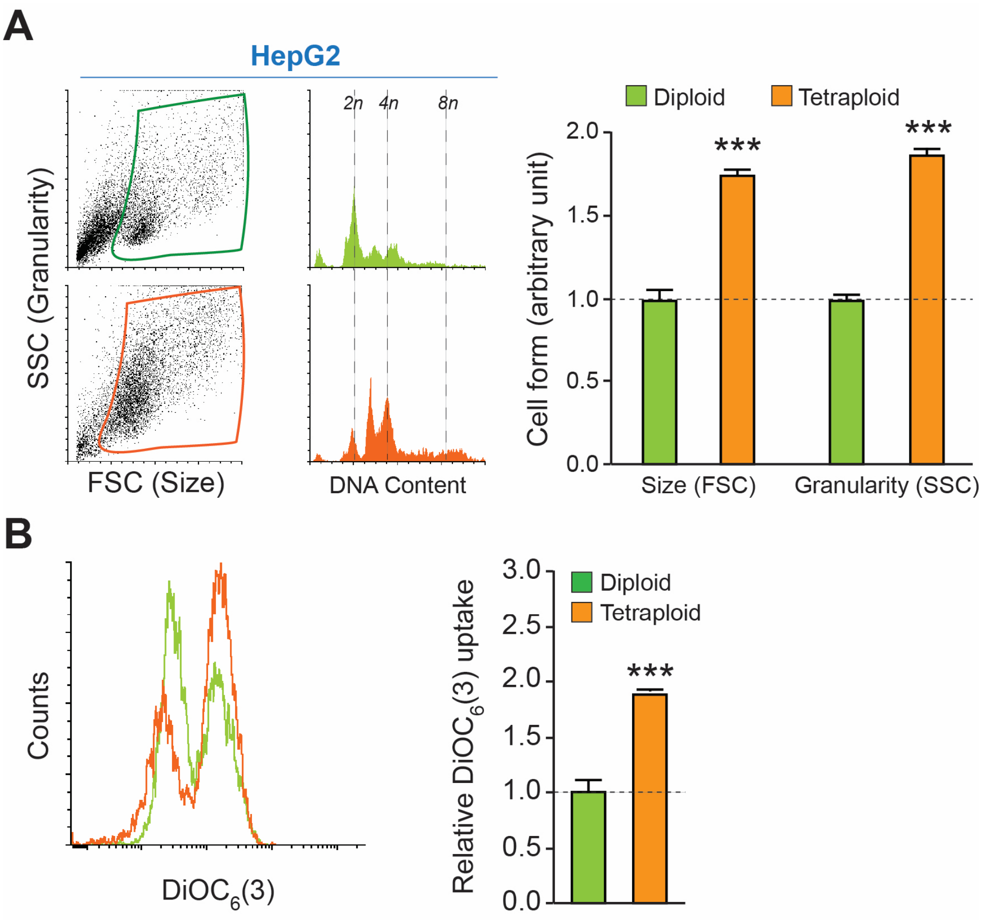

Figure S2: Characterization of the size, form and mitochondrial potential of diploid and tetraploid liver cancer cells. A. Flow cytometry dot plots showing forward scatter (size) versus side scatter (granularity) and the associated histograms showing the cell cycle of near-diploid and near-tetraploid HepG2 cells. Related quantification data is also shown. **B.** Near-diploid and near-tetraploid HepG2 cells were stained with the dye DiOC6(3) to quantify the mitochondrial transmembrane potential (ΔΨ_m) by flow cytometry. The histograms show overlap between the near-diploid and near-tetraploid conditions, while the quantification data reveal mitochondrial uptake of the dyes in both conditions. Diploid cells are labeled in green and tetraploid cells in orange. Data are reported as means ± SEM (n ≥ 3). *** p < 0.001 (Mann–Whitney test) compared with diploid cells.
